# Characterization of the TRBP domain required for Dicer interaction and function in RNA interference

**DOI:** 10.1186/1471-2199-10-38

**Published:** 2009-05-07

**Authors:** Sylvanne M Daniels, Carlos E Melendez-Peña, Robert J Scarborough, Aïcha Daher, Helen S Christensen, Mohamed El Far, Damian FJ Purcell, Sébastien Lainé, Anne Gatignol

**Affiliations:** 1 Virus-Cell Interactions Laboratory, Lady Davis Institute for Medical Research, 3999 Côte Ste Catherine, Montréal, Québec, H3T1E2, Canada; 2 Department of Microbiology and Immunology, McGill University, Montréal, Québec, Canada; 3 Department of Experimental Medicine, McGill University, Montréal, Québec, Canada; 4 Department of Microbiology and Immunology, University of Melbourne, Parkville, Australia; 5 Current address: GSK-Biological, Laval, QC, Canada; 6 Current address: Centre de Recherche du CHUM, Hôpital Saint-Luc, Montréal, QC, Canada; 7 Current address: CNRS UMR 5097, Université de Bordeaux 2, Bordeaux, France

## Abstract

**Background:**

Dicer, Ago2 and TRBP are the minimum components of the human RNA-induced silencing complex (RISC). While Dicer and Ago2 are RNases, TRBP is the double-stranded RNA binding protein (dsRBP) that loads small interfering RNA into the RISC. TRBP binds directly to Dicer through its C-terminal domain.

**Results:**

We show that the TRBP binding site in Dicer is a 165 amino acid (aa) region located between the ATPase and the helicase domains. The binding site in TRBP is a 69 aa domain, called C4, located at the C-terminal end of TRBP. The TRBP1 and TRBP2 isoforms, but not TRBPs lacking the C4 site (TRBPsΔC4), co-immunoprecipitated with Dicer. The C4 domain is therefore necessary to bind Dicer, irrespective of the presence of RNA. Immunofluorescence shows that while full-length TRBPs colocalize with Dicer, TRBPsΔC4 do not. *tarbp2*^-/- ^cells, which do not express TRBP, do not support RNA interference (RNAi) mediated by short hairpin or micro RNAs against EGFP. Both TRBPs, but not TRBPsΔC4, were able to rescue RNAi function. In human cells with low RNAi activity, addition of TRBP1 or 2, but not TRBPsΔC4, rescued RNAi function.

**Conclusion:**

The mapping of the interaction sites between TRBP and Dicer show unique domains that are required for their binding. Since TRBPsΔC4 do not interact or colocalize with Dicer, we suggest that TRBP and Dicer, both dsRBPs, do not interact through bound dsRNA. TRBPs, but not TRBPsΔC4, rescue RNAi activity in RNAi-compromised cells, indicating that the binding of Dicer to TRBP is critical for RNAi function.

## Background

RNA interference (RNAi) is a natural mechanism used by eukaryotes for gene silencing. This mechanism uses small double-stranded (ds)RNA, named micro (mi) or small interfering (si) RNAs, which are complementary to a target gene to degrade the corresponding mRNA or block its translation. The dsRNA triggers the assembly of a ribonucleoprotein complex called the RNA-induced silencing complex (RISC) [1]. The mechanism and complex composition has been best studied in *Drosophila melanogaster*. This is an enzymatic process that involves RNAse III-like proteins (Dicer and Drosha) and a dsRNA binding protein (dsRBP; R2D2 and Loquacious) [2-4]. The second step, which leads to the cleavage of the target mRNA, includes an Argonaute (Ago) protein [5,6].

Mammalian cells have a single Dicer protein, with a molecular weight of ~200 kDa. Dicer contains an ATPase/RNA helicase domain, a DUF domain, a PAZ domain, two RNase III domains and a dsRNA binding domain (dsRBD) [5]. The Dicer PAZ domain associates with the PIWI domain of Ago2 [7]. Dicer is responsible for cleaving the dsRNA trigger (miRNA or siRNA) so it can be loaded into the RISC. Ago2 is then recruited to the RISC where it cleaves the target mRNA or mediates translation inhibition after its association with the complementary strand from the mi/siRNA [8]. Dicer knock-out (Dcr^-/-^) mice and cells are not viable indicating a major function for this protein during development and normal cell function [9]. In human cells, the dsRBP that associates with Dicer is the TAR RNA binding protein, TRBP. This protein is required for RNAi function mediated by both siRNAs and miRNAs [10-12], where it acts as a biosensor in the choice of dsRNA loaded into the RISC [13,14]. Furthermore, Dicer, TRBP and Ago2 are necessary and sufficient for *in vitro *reconstitution of RNAi activity [15].

TRBP1 and TRBP2 are isoforms of the cellular protein TRBP which was isolated by its ability to bind the human immunodeficiency (HIV)-1 TAR RNA and characterized for its stimulation of the expression of the HIV long terminal repeat in human and murine cells [16-20]. TRBPs have two dsRBDs, a KR-helix motif within dsRBD2 and a Medipal domain that mediates protein-protein interactions [21-25]. TRBPs also bind to PKR and PACT through their dsRBDs and to Merlin, Dicer and PACT through their Medipal domain [11,17,25-27]. TRBPs are encoded by the *tarbp2 *gene. Two adjacent promoters initiate transcription of alternative first exons for TRBP1 and TRBP2 mRNAs and as a consequence, in comparison to TRBP1, TRBP2 has 21 additional amino acids (aa) [28,29]. TRBPs have functional activities in spermatogenesis, cell growth, oncogenesis and viral replication linked to their RNA- and protein-binding abilities [27,30-32]. Among these, the TRBP-Dicer interaction and its function as part of the RISC has been identified as an important component of the RNAi pathway. In this paper, we further characterize the specific domains in TRBP and Dicer that are required for their interaction and we analyze the consequences of this interaction in RNAi function.

## Results

### TRBP Medipal domain interacts with Dicer through a unique domain in Dicer located between the ATPase and the helicase motifs

**Figure 1:**
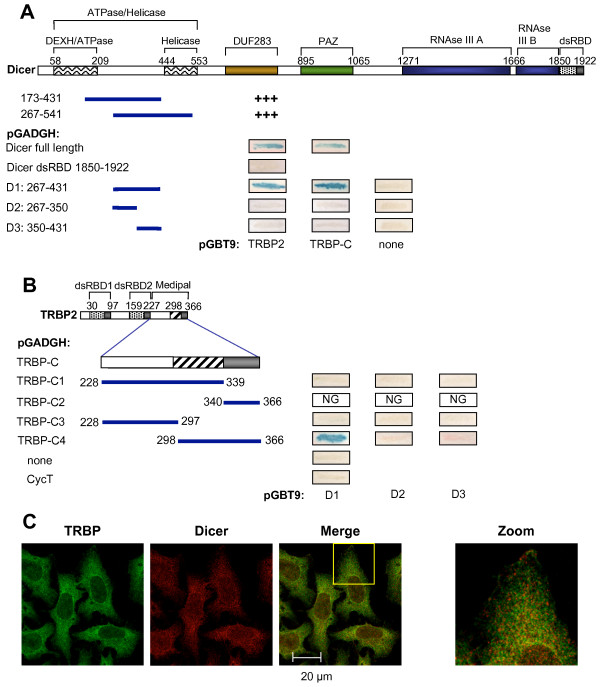
**Determination of Dicer-TRBP interaction domains and colocalization**. **A) Dicer interacting domain is located in a unique region between the ATPase and the helicase domains**. (Top) Schematic representation of Dicer protein and its domains. (Bottom) Dicer 173–431 or 267–541 represent the isolated interacting Dicer clones in a two-hybrid screen using TRBP as bait. Yeast two hybrid assay using Dicer or Dicer domains expressed in pGADGH vector and their interactions with TRBP2 clones expressed in pGBT9 vector. **B) The Medipal subdomain in TRBP is located between aa 298 and 366**. (Top) Schematic representation of TRBP2 protein and its domains. (Bottom) Yeast two hybrid assay using TRBP2 or TRBP domains expressed in pGADGH vector and their interactions with Dicer domains identified in A and expressed in pGBT9 vector. NG means no growth. Shown are representative assays of three independent experiments. Dicer and TRBP2 schematic representation of full-length proteins are at scale. **C) Endogenous TRBP and Dicer colocalize**. HeLa cells were fixed and labeled with rabbit anti-TRBP673 and mouse anti-Dicer (mAb42) followed by anti-rabbit Alexa Fluor 488 and anti-mouse Alexa Fluor 546. The cells were mounted and assayed for fluorescence by laser scanning confocal microscopy on a Zeiss LSM Pascal 5 microscope. Alexa Fluor 488 and Alexa Fluor 546 fluorescences are represented as green and red, respectively, while merged colors appear as yellow. Images showing the colocalization of the two proteins detected by the yellow dots are amplified on the right.

The TRBP-Dicer interaction was found by immunoprecipitation (IP) with a Dicer antibody followed by mass spectrometry analysis of the interacting proteins [10,11]. We independently identified Dicer in a two-hybrid screen using TRBP as bait. This screen resulted in the identification of six clones that interacted with TRBP. Among these, the only clones which corresponded to an RBP mapped to Dicer aa 173–431 or aa 267–541 (Fig. 1A). Because both TRBP and Dicer are dsRBPs, and these corresponding domains often dimerize [17,25], we also verified if the Dicer dsRBD (aa 1850–1922) could interact with TRBP, but no interaction was found. We next expressed the common sequence between the isolated two-hybrid clones, aa 267–431 called D1, on pGBT9 and showed its interaction with TRBP2 or TRBP C (Medipal domain) expressed in pGADGH. This domain was further divided in two halves expressing aa 267–350 and 350–431. Neither domain interacted with TRBP, indicating that the 165 aa D1 domain is the smallest TRBP-interacting domain defined in this assay.

The Medipal domain was previously defined as a protein-protein interacting domain in TRBP (aa 228–366 or TRBP-C) that binds Merlin, Dicer and PACT [25]. TRBP interaction with PACT in this domain has been mapped to aa 287–366 in TRBP2. To determine if TRBP-Dicer interaction can be limited to a shorter domain, we first expressed the subdomains as C1 and C2 expressing aa 228–339 and 340–366 respectively (Fig. 1B). C1 did not show an interaction and the expression of C2 repeatedly induced yeast growth arrest indicating a cytotoxicity of this domain. The domain was next divided into C3 and C4 expressing aa 228–297 and 298–366 respectively. Only C4 showed a specific interaction with D1, indicating that this subdomain of the Medipal is the active component for Dicer binding.

The cellular distribution of Dicer and TRBP has not yet been fully elucidated. We next examined their localization in the cell using immunofluorescence with antibodies directed against endogenous TRBP and Dicer. Interestingly, TRBP localized mainly to the cytoplasm, while Dicer was distributed throughout the cytoplasm and the nucleus. We found significant colocalization between Dicer and TRBP in HeLa cells predominantly in the perinuclear space (Fig. 1C). These data and previous results confirm that TRBP and Dicer can interact in a physiological environment.

### The C4 sequence within the Medipal domain of TRBP is necessary and sufficient to bind Dicer in mammalian cells

**Figure 2:**
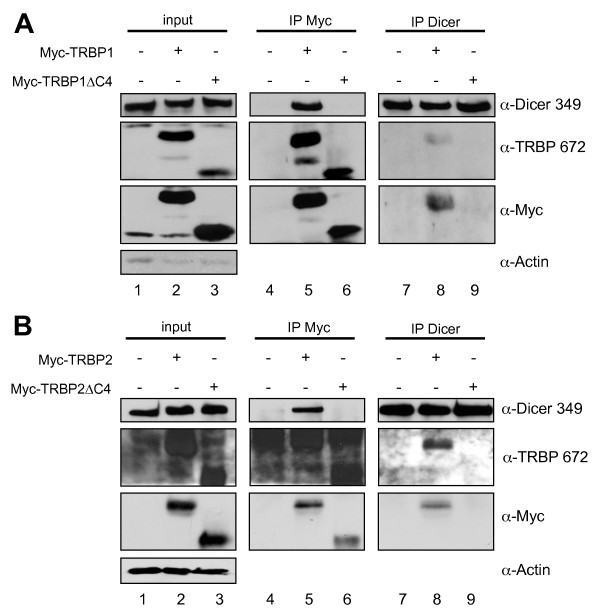
**The C4 domain is necessary and sufficient to mediate TRBP-Dicer interaction**. **A) TRBP1 or TRBP1ΔC4-Dicer interactions by immunoprecipitation**. *tarbp2*^-/- ^cells were transfected with none (lanes 1, 4, 7) or 15 μg of pCMV-Myc-TRBP1 (lanes 2, 5, 8), pCMV-Myc-TRBP1ΔC4 (lanes 3, 6, 9). IP was performed with 1 mg of protein and anti-Myc antibody (lanes 4–6) or anti-Dicer antibody (lanes 7–9). 100 μg of proteins from each lysate (input; lanes 1–3) and the immunoprecipitated complexes (lanes 4–9) were run on a 7.5% SDS-PAGE and blotted using anti-Dicer, anti-TRBP 672, anti-Myc and anti-actin antibodies. The lower bands in lanes 1 and 2 correspond to the migration front. **B) TRBP2 or TRBP2ΔC4-Dicer interactions by immunoprecipitation**. *tarbp2*^-/- ^cells were transfected with none (lanes 1, 4, 7) or 15 μg of pCMV-Myc-TRBP2 (lanes 2, 5, 8), pCMV-Myc-TRBP2ΔC4 (lanes 3, 6, 9). IP was performed with 1 mg of protein and anti-Myc antibody (lanes 4–6) or anti-Dicer antibody (lanes 7–9). 100 μg of proteins from each lysate (input; lanes 1–3) and the immunoprecipitated complexes (lanes 4–9) were run on a 7.5% SDS-PAGE and blotted using anti-Dicer, anti-Myc and anti-actin antibodies.

To determine the role of the C4 domain in the protein context, we used a complete TRBP knock-down environment. Using murine tail embryonic fibroblasts (TEFs) *tarbp2 *knock-out cells [26,32], we evaluated the importance of the C4 domain in TRBP on the interaction between TRBP and Dicer. We expressed TRBP1 and TRBP2 as Myc-tagged proteins as previously [25], and Myc-TRBP1ΔC4 and Myc-TRBP2ΔC4 lacking this domain. TRBPsΔC4 were tested for their binding to Dicer by IP (Fig. 2). In conditions where Myc-TRBP1 interacts with endogenous Dicer, Myc-TRBP1ΔC4 did not interact with Dicer, whether the IP was performed with an anti-Myc or an anti-Dicer antibody (Fig. 2A). Results with Myc-TRBP2 and Myc-TRBP2ΔC4 were similar (Fig. 2B). We have previously excluded a binding of TRBP-Dicer through the dsRBDs in TRBP or through RNA [11]. The loss of TRBP-Dicer interaction in the absence of the C4 domain, but in the presence of RNA, further argues against the involvement of RNA in the interaction. These combined results (Figs. 1 and 2) indicate that the C4 domain is necessary and sufficient to mediate the interaction with Dicer and that no other cellular protein or RNA can rescue the lack of interaction between the mutant TRBP and Dicer in mammalian cells.

### TRBP, but not TRBPΔC4 colocalizes with Dicer in cells

**Figure 3:**
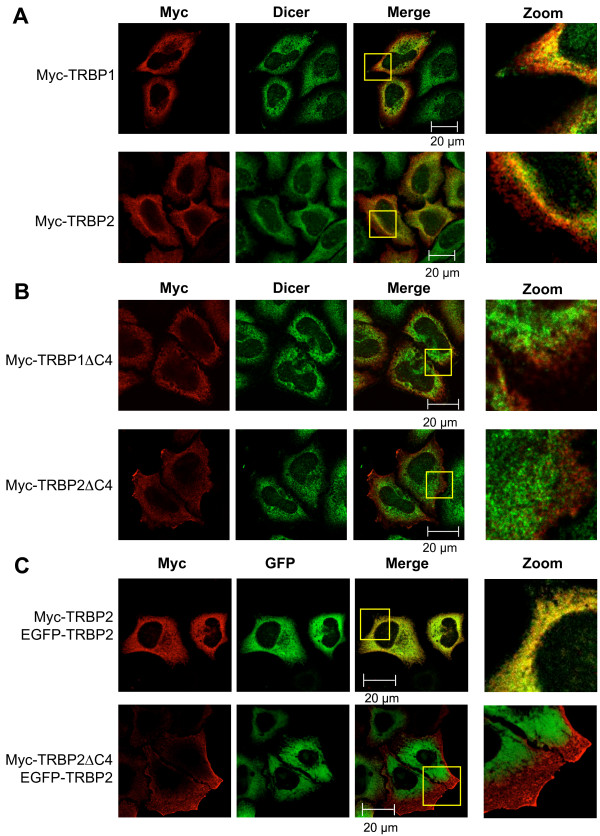
**C4 domain in TRBP is required for colocalization with endogenous Dicer**. HeLa cells were transfected with 0.5 μg of pCMV-Myc-TRBP1 or pCMV-Myc-TRBP2 (**A**), pCMV-Myc-TRBP1ΔC4 or pCMV-Myc-TRBP2ΔC4 (**B**), as indicated on the left. Cells were fixed and labeled with mouse anti-Myc and rabbit anti-Dicer (pAb349) followed by anti-rabbit Alexa Fluor 488 and anti-mouse Alexa Fluor 546. The cells were mounted and assayed for fluorescence by laser scanning confocal microscopy on a Zeiss LSM Pascal 5 microscope. Alexa Fluor 488 and Alexa Fluor 546 fluorescences are represented as green and red, respectively, while merged colors appear as yellow. Images showing the colocalization of the two proteins detected by the yellow dots or absence of colocalization are amplified on the right. **C) TRBP2 and TRBP2ΔC4 do not colocalize**. HeLa cells were transfected with 0.5 μg pEGFP-C1-TRBP2 and either pCMV-Myc-TRBP2 or pCMV-TRBP2ΔC4. Cells were fixed and labeled with anti-Myc followed by anti-mouse Alexa Fluor 546. The cells were mounted and assayed as previously. EGFP signal is shown in green and Alexa Fluor 546 fluorescence is represented in red. Images showing colocalization of the two proteins detected by yellow dots, or absence of colocalization, are amplified of the right.

To set up conditions to compare the cellular distribution of wild-type and truncated TRBPs, we first expressed TRBP1 and TRBP2 Myc-tagged proteins in HeLa cells. The cellular distribution of the Myc-tagged proteins, as well as their colocalization with Dicer was consistent with the endogenous localization of TRBP (Fig. 3A compared with 1C). Importantly, we found the tagged TRBP constructs co-localized with Dicer in the perinuclear space.

To further define the importance of the C4 subdomain within TRBP Medipal, we expressed Myc-TRBP1ΔC4 and Myc-TRBP2ΔC4 in the same conditions as the wild type proteins (Fig. 3B). With these mutants, we observed a marked change in cellular distribution as compared to the wild-type proteins. Of importance, the mutant proteins did not colocalize with Dicer in the perinuclear space and for TRBP2ΔC4, a clear relocalization to the plasma membrane was observed. These results emphasize the importance of the C4 subdomain within the Medipal domain as a major determinant in the cellular distribution of TRBP.

To further verify the distributions of full-length TRBP2 as compared to TRBP2ΔC4, their localization was examined in relation to an EGFP-tagged TRBP2 construct. EGFP-TRBP2 and Myc-TRBP2 or Myc-TRBP2ΔC4 were coexpressed as previously in HeLa cells. The colocalization of the EGFP-tagged form of TRBP2 with the Myc-tagged form of TRBP2 is shown by an extensive yellow coloration in the cytoplasm (Fig. 3C). In comparison, colocalization of Myc-TRBP2ΔC4 with EGFP-TRBP2 was significantly reduced, mainly due to a shift of the truncated protein to the cellular membrane. This further supports the observation that the cellular distribution of truncated TRBP2 significantly differs from that of the full-length protein.

### TRBP is required for RNA interference mediated by shRNA or exogenous miRNA against EGFP in murine cells

**Figure 4:**
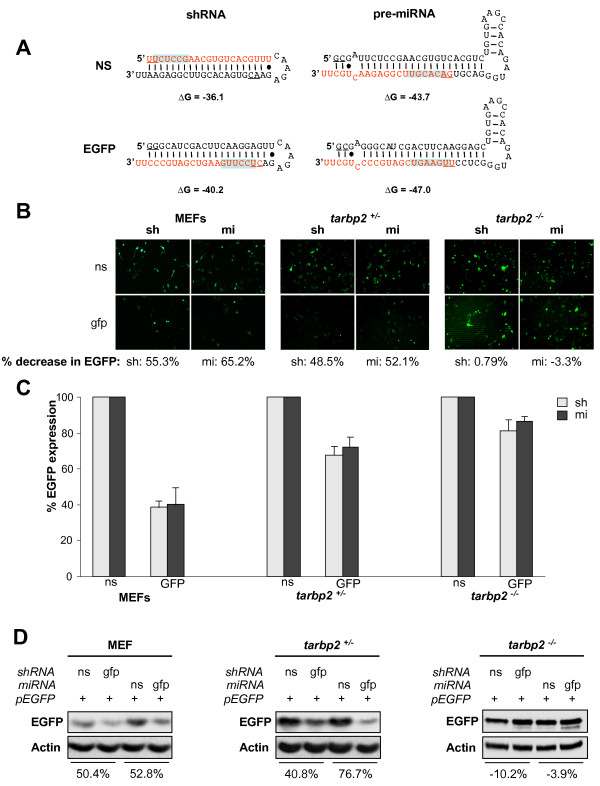
**TRBP is required for RNA interference mediated by shRNA or exogenous miRNA against EGFP in murine cells**. **A) Schematic representation of the sh and miRNAs transcribed from pCRII-U6+27 vectors and targeting non specific (ns) or EGFP mRNA**. The guide strand is shown in red. The seed sequence is highlighted in blue. **B, C, D) sh and miRNA against EGFP in murine cells are active only when TRBP is present**. MEFs (left panels), TEFs heterozygous for TRBP expression (*tarbp2*^+/-^; middle panels) or TEFs disrupted for TRBP expression (*tarbp2*^-/-^; right panels) were transfected with 1 μg of EGFP-C1 and 4 μg of ns or anti-EGFP sh- or mi-RNAs, as indicated. **B) **Fluorescence of live cells was monitored on an inverted microscope and a representative picture is shown. Percentages reflect knock-down of EGFP expression as calculated by mean luminosity intensity. **C) **Cells were analyzed by fluorescence automated cell sorting (FACS). Reporter expression is calculated as the percentage of EGFP expression in the presence of sh- or miRNA compared to ns normalized to 100%. Values are means of four independent experiments ± SEM. **D) **Total cell extracts were analyzed by immunoblot for EGFP and actin expression. Percent decrease of EGFP signal was calculated by densitometry analysis.

The requirement for TRBP in the RNAi pathway has been shown using cells that have been partially knocked down for TRBP using siRNAs [10,11]. To determine RNAi activity in a complete TRBP knock-down environment, we used *tarbp2*^-/- ^cells previously described [26] compared to *tarbp2*^+/- ^and wild type murine embryo fibroblasts (MEF). To have a qualitative and a quantitative assay, we designed shRNAs against EGFP, and exogenous miRNAs that have the siRNA sequence against EGFP in an miR30 backbone, as previously shown for another target [33] (Fig. 4A). Although these constructs were both active in MEF and *tarbp2*^+/- ^cells as shown by EGFP fluorescence, no significant activity could be detected in *tarbp2*^-/- ^cells (Fig. 4B). When quantified by flow cytometry, the activity of sh-EGFP and mi-EGFP was 62 and 60% in MEF, 32 and 28% in *tarbp2*^+/- ^cells but only 18 and 14% in *tarbp2*^-/- ^cells (Fig. 4C). To quantify RNAi activity by another method, a Western blot analysis was performed and showed no inhibition of EGFP expression by either sh- or miRNAs in *tarbp2*^-/- ^cells (Fig. 4D).

### TRBP1 and TRBP2, but not TRBPsΔC4 can rescue the RNAi pathway in tarbp2^-/- ^cells

**Figure 5:**
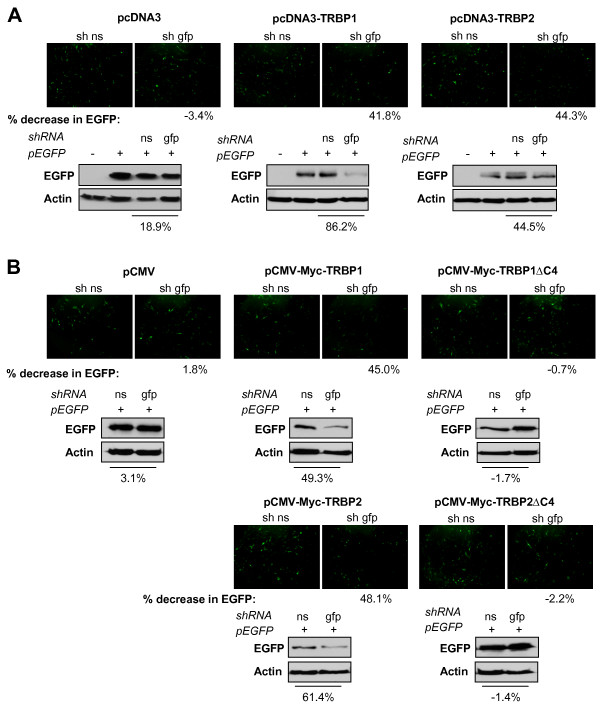
**TRBP1 and TRBP2, but not TRBPsΔC4 rescue the RNAi pathway in *tarbp2*^-/- ^cells**. **A) TRBP1 and TRBP2 rescue the RNAi pathway in *tarbp2*^-/-^cells**. *tarbp2*^-/- ^cells were transfected with none or 1 μg of EGFP and 4 μg of non-silencing (ns) or anti-EGFP (gfp) shRNA as indicated, and 1 μg of pcDNA3 (left panels), pcDNA3-TRBP1 (middle panels) or pcDNA3-TRBP2 (right panels). (Top panels) EGFP knock-down was assessed by fluorescence, and average percentages of EGFP knockdown were calculated from luminosity intensity values of four fields per condition. Representative photos are shown. (Bottom panels) Cell extracts were analyzed by immunoblotting against EGFP and actin. Percent decrease of EGFP signal was calculated by densitometry analysis. **B) TRBP1ΔC4 and TRBP2ΔC4 do not rescue the RNAi pathway in *tarbp2*^-/- ^cells**. *tarbp2*^-/- ^cells were transfected with 1 μg of EGFP and 4 μg of non-silencing (ns) or anti-EGFP (gfp) shRNA as indicated, and 1 μg of either pCMV, pCMV-Myc-TRBP1, pCMV-Myc-TRBP1ΔC4, pCMV-Myc-TRBP2 or pCMV-Myc-TRBP2ΔC4 as indicated. (Top panels) EGFP knock-down was assessed by fluorescence, and average percentages of EGFP knockdown were calculated from luminosity intensity values of four fields per condition. Representative photos are shown. (Bottom panels) Cell extracts were analyzed by immunoblotting against EGFP and actin. Percent decrease of EGFP signal was calculated by densitometry analysis.

To further determine if TRBP could rescue RNAi in *tarbp2*^-/- ^murine cells, pcDNA3 or pcDNA3 constructs expressing TRBP1 or TRBP2 were cotransfected with EGFP and shRNA against EGFP and verified for cell fluorescence and protein expression by Western blot (Fig. 5). Consistent with results in Fig. 4D, pcDNA3 empty vector did not restore the activity of the shRNA in *tarbp2*^-/- ^cells. In contrast, we observed that RNAi activity was recovered when TRBP1 or, to a lesser extent, TRBP2, were expressed in the cells (Fig. 5A). We next transfected the same cells with Myc-TRBP1, Myc-TRBP2 and their ΔC4 counterparts (Fig. 5B). The full-length TRBP proteins rescued the RNAi function but the ΔC4 mutants did not, indicating that binding of the C4 domain in TRBP to the D1 domain in Dicer is essential for efficient RNAi activity.

### TRBP is required for RNAi mediated by shRNA or exogenous miRNA against EGFP in human cells

**Figure 6:**
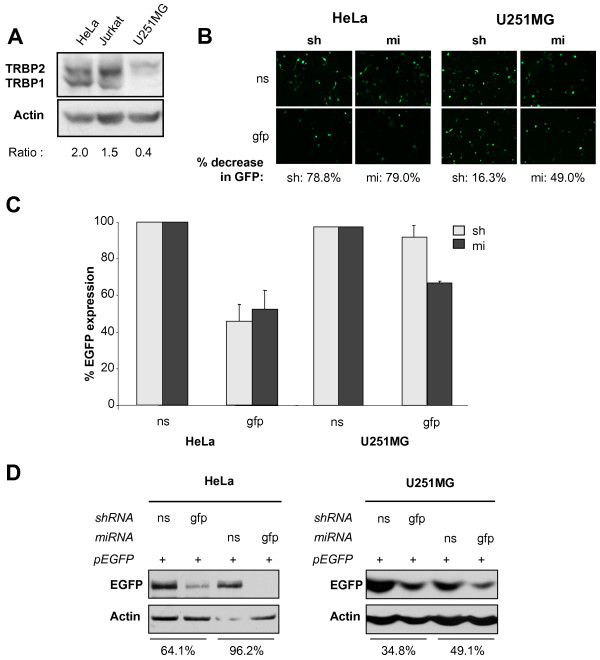
**TRBP is required for RNA interference mediated by shRNA or exogenous miRNA against EGFP in human cells**. **A) TRBP expression in human cells**. Expression levels of TRBP was assessed in HeLa, Jurkat and U251MG cells using immunoblotting. The TRBP:Actin ratio calculated by densitometry is indicated below each lane. **B, C, D) sh- and miRNA against EGFP are less active in astrocytes than in HeLa cells**. HeLa (left) or astrocytes U251MG (right) cells were transfected with 1 μg of EGFP-C1 and 4 μg of ns or anti-EGFP sh- or miRNAs as indicated. **B) **Cellular fluorescence of live cells was monitored on an inverted microscope and a representative picture is shown. Percentages reflect knock-down of EGFP expression as calculated by mean luminosity intensity. **C) **Cells were analyzed by FACS. Reporter expression is calculated as the percentage of EGFP expression in the presence of sh or miRNA compared to ns normalized to 100%. Values are means of four independent experiments ± SEM. **D) **Total cell extracts were analyzed by immunoblot for EGFP and actin expression. Percent decrease of EGFP signal was calculated by densitometry analysis.

Thus far, all human cell lines described express TRBP, but we have shown previously that U251MG, an astrocytoma cell line, expresses an undetectable amount of TRBP1 and a decreased amount of TRBP2 compared to HeLa or Jurkat cells [28,29] (Fig. 6A). To get more insight into the importance of TRBP in human cell lines, we used the previous assays to evaluate RNAi function in the relative absence of TRBP. By fluorescence analysis, we observed no decrease of EGFP expression with the sh-EGFP constructs and a weaker decrease of EGFP with the mi-EGFP constructs in the U251MG cells compared to HeLa cells (Fig. 6B). Quantification of this knock-down supported our initial observations. To quantify these observations differently, we performed flow cytometry analysis and found similar differences with 57 and 45% decrease in HeLa, but only 6 and 32% decrease in U251MG for sh- and mi-EGFP, respectively (Fig. 6C). Similarly, Western blots indicated that U251MG cells poorly support RNAi activity mediated by shRNAs and mildly support RNAi mediated by miRNAs compared to HeLa cells (Fig. 6D).

### TRBP1 and TRBP2, but not TRBPsΔC4, can rescue the RNAi pathway in human U251MG cells

**Figure 7:**
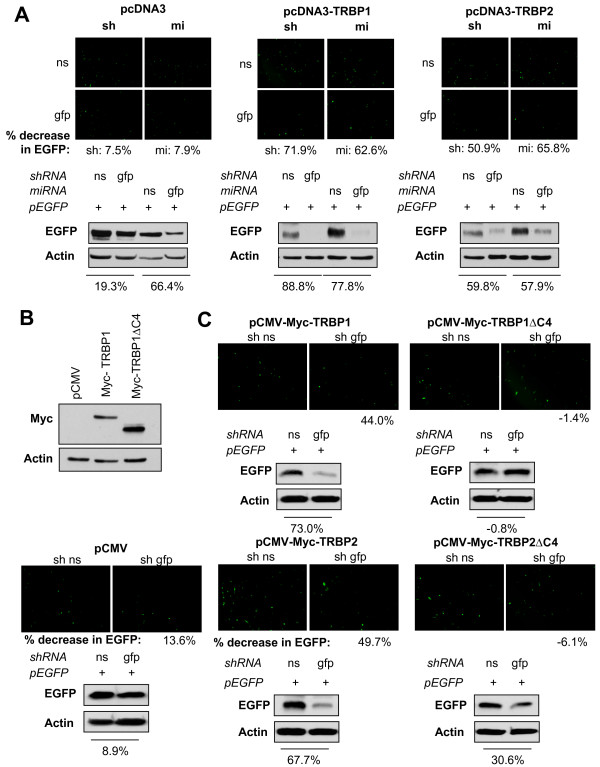
**TRBP1 and TRBP2, but not TRBPsΔC4 rescue the RNAi pathway in U251MG cells**. **A) TRBP1 and TRBP2 rescue the RNAi pathway in U251MG cells**. Human U251MG cells were transfected with none or 1 μg of EGFP and 4 μg of either non-silencing (ns) or anti-GFP (gfp) sh- or miRNA, as indicated, and 1 μg of pcDNA3 (left panels), pcDNA3-TRBP1 (middle panels) or pcDNA3-TRBP2 (right panels). (Top panels) EGFP knock-down was assessed by fluorescence, and average percentages of EGFP knockdown were calculated from luminosity intensity values of four fields per condition. Representative photos are shown. (Bottom panels) Cell extracts were analyzed by immunoblotting against EGFP and actin. Percent decrease of EGFP signal was calculated by densitometry analysis. **B) Myc-TRBP1 and TRBP1ΔC4 are equally expressed in U251MG cells**. U251MG cells were transfected with 1 μg of either pCMV, pCMV-Myc-TRBP1 or pCMV-Myc-TRBP1ΔC4 as indicated. Cell extracts were analyzed by immunoblotting against Myc and actin. **C) TRBP1ΔC4 and TRBP2ΔC4 do not rescue the RNAi pathway in U251MG cells**. U251MG cells were transfected with 1 μg of EGFP and 4 μg of non-silencing (ns) or anti-EGFP (gfp) shRNA as indicated, and 1 μg of either pCMV, pCMV-Myc-TRBP1, pCMV-Myc-TRBP1ΔC4, pCMV-Myc-TRBP2 or pCMV-Myc-TRBP2ΔC4 as indicated. (Top panels) EGFP knock-down was assessed by fluorescence, and average percentages of EGFP knockdown were calculated from luminosity intensity values of four fields per condition. Representative photos are shown. (Bottom panels) Cell extracts were analyzed by immunoblotting against EGFP and actin. Percent decrease of EGFP signal was calculated by densitometry analysis.

To further confirm the role of TRBP in RNAi activity in human cells, U251MG cells were complemented with either pcDNA3, pcDNA3-TRBP1 or pcDNA3-TRBP2 constructs. Their ability to perform RNAi was assessed as previously. As in murine *tarbp2*^-/- ^cells, TRBPs were able to effectively restore RNAi activity in these cells (Fig. 7A), with TRBP1 being more efficient than TRBP2. pCMV-Myc-TRBP1 and pCMV-Myc-TRBP2 constructs were equally effective in restoring RNAi activity, however ΔC4 mutants were unable to do so despite a similar expression (Fig. 7B, C). These results confirm that the Dicer-binding domain of TRBP is required for efficient RNAi activity in human cells.

## Discussion

The minimal active human RISC is composed of three proteins, Dicer, TRBP and Ago2 [5,15]. Within this complex, Dicer and Ago2 have RNase activities, whereas TRBP is important in loading the appropriate dsRNA into the complex [13,14]. Other proteins have been shown to associate with the RISC, however their roles remain to be fully determined [34]. We further defined the requirement for TRBP in RNAi using murine cells that are completely deficient for TRBP (*tarbp2*^-/-^). Using an shRNA and an miRNA targeting EGFP, these cells displayed low or no RNAi activity as compared to murine cells expressing TRBP (*tarbp2*^+/- ^and MEF) (Fig. 4), confirming that TRBP is an essential protein for RNAi activity in these cells. The different approaches used to assess RNAi activity yielded slightly different intensities of EGFP knock-down, which can be attributed to the different sensitivities of each technique. Because no human cell has been shown to have a complete TRBP deficit, we evaluated the RNAi function in astrocytes, which have a weak expression of TRBP. This correlated with a low and moderate activity for the sh- and miRNAs, respectively, compared to HeLa cells (Fig. 6). In this case, the difference in RNAi activity between U251MG and HeLa cells follows the same trend in fluorescence, FACS and western blotting. The ability of U251MG cells to knock-down GFP expression using miRNA-based silencing is likely explained by the residual TRBP2 expression in this cell type. Our results demonstrate that in both murine and human cells, TRBP is required for efficient RNAi activity.

The interaction between Dicer and TRBP has been shown to be direct *in vitro *and *in vivo *with no requirement for ssRNA, dsRNA or another protein [11]. Although the site of interaction in TRBP was determined to be in the C-terminal end, no distinct functional domain had been defined for Dicer [11]. Two-hybrid assays further defined these domains as aa 267–431 (called D1) in Dicer and aa 298–366 in TRBP2 (called C4) (Fig. 1). D1 is located between the ATPase and the helicase domains in Dicer. It was shown previously that ATP is not required for Dicer function in RNAi [35,36], suggesting that either this domain is not functional in the human protein or that it plays a role in other processes. Interestingly, although a complete knock-down of Dicer is lethal, cells defective for only the ATPase/helicase domain are viable with only a defect in miRNA biogenesis [9,37]. Here we show that the functional importance of the ATPase/helicase domain is in supporting the interaction with TRBP. These combined results suggest that the ATPase/helicase domain is not functional in the human protein, and that the sole function of this region may be to recruit TRBP through the D1 domain. Interestingly, when searched in BLASTP, D1 has no homology with any other protein in the databases, showing its unique properties.

Further demonstration that the C4 domain is solely responsible for TRBP binding to Dicer is shown by the IP assays in the presence of complete cell extracts from *tarbp2*^-/- ^cells (Fig. 2). In this assay, neither TRBP1ΔC4, nor TRBP2ΔC4, immunoprecipitated with Dicer. Together with previous results that showed a direct interaction between TRBP and Dicer in the absence of RNA or other proteins, these results confirm that no bridge between Dicer and TRBP occurs *via *dsRNA or another protein. TRBPsΔC4 and Dicer both have functional dsRBDs that are able to bind dsRNA. The absence of interaction between the TRBP mutants and Dicer suggests that they do not bind the same RNA at the same time.

Dicer and TRBP are cytoplasmic proteins but their colocalization has not been studied. Here, we show that endogenous Dicer can be observed in both the nucleus and the cytoplasm, while TRBP is distributed predominantly in the cytoplasm (Fig. 1C). Colocalization of the two proteins was found to occur predominantly in the perinuclear space. Overexpression of TRBPs did not affect their cellular distribution as immunofluorescence of Myc-tagged constructs revealed similar staining to that of the endogenous protein (Fig. 1C compared to 3A). As neither Myc-TRBP1ΔC4, nor Myc-TRBP2ΔC4 colocalized with Dicer in the perinuclear space, we conclude that binding of TRBP to Dicer is a major determinant of TRBP cellular distribution and our results suggest that RNAi takes place in the perinuclear space. In contrast to Myc-TRBP1ΔC4, Myc-TRBP2ΔC4 was partially associated to the cell membrane. Because TRBP2 has only 21 more aa in its N-terminus than TRBP1, these aa might be responsible for the membrane association, which is only revealed when its interaction with Dicer is abolished. A closer analysis of the 21 aa sequence and the protein's posttranslational modifications will be necessary to elucidate this function.

This slight difference between TRBP1 and TRBP2 also correlates with the rescue of the RNAi function mediated by shRNAs against EGFP (Figs. 5A and 7A). In these assays, TRBP1 completely rescued the shRNA activity while TRBP2 rescued it partially. In contrast, we could not rescue shRNA activity using the ΔC4 mutants (Figs. 5B and 7C). This demonstrates that the C4 domain, which mediates TRBP interaction with Dicer, is also required for RNAi activity to occur. These results are compatible with a role of Dicer in the recognition and cleavage of precursor miRNA and for TRBP in loading small siRNAs into the RISC [5,13,14]. This study provides new insights into the interaction between TRBP and Dicer in the RISC. These results open the way for further studies into differences between the specific roles of TRBP1 and TRBP2 in the RNAi pathway.

## Conclusion

This is the first study that maps Dicer and TRBP interaction domains precisely to 165 aa and 69 aa regions, respectively. In Dicer, the motif is located in a unique region between the ATPase and the helicase domains. In murine *tarbp2*^-/- ^cells and in U251MG astrocytoma cells that do not support and partially support RNAi, respectively, the TRBP1 and TRBP2 isoforms can rescue this pathway, whereas mutant forms lacking the interacting domain cannot. TRBP1 and TRBP2 colocalize and immunoprecipitate with Dicer whereas the mutants cannot, suggesting that TRBP and Dicer do not bind to the same RNA at the same time during the RNAi process.

## Methods

### Plasmid constructions

pGADGH-Dicer, pGADGH and pGBT9 TRBP and TRBP C have been described [11]. pGADGH-Cyclin T1 has been described [38]. TRBP-C fragments (encoding C1: aa 228–339, C2: aa 340–366, C3: aa 228–297, C4: aa 298–366) were generated from pcDNA3-TRBP2 [18] by PCR. PCR products were cut by *Xma*I and *Xho*I and subcloned in frame into the yeast expression vector pGADGH [17]. The sequences of the primers used are listed below:

TRBP C1 sense: 5'-TCCCCCCGGGGGTGCCTCTGGATGCACGGGATGG-3'

TRBP C1 antisense: 5'-CCAGCTCTCGAGTCACTTGCTGCCTGC-3'

TRBP C2 sense: 5'-CCACTGTCCCGGGAATGTCTGCAACCACC-3'

TRBP C2 antisense: 5'-CCTGGTCTCGAGCTAGCCATGACACAC-3'

TRBP C3 antisense: 5'-CCTCAGACTCGAGCTAGAGGACACGGC-3'

TRBP C4 sense: 5'-CTGCCGCCCGGGAATGGAGCTCTCTGAG-3'

TRBP C1 sense and TRBP C3 antisense primers were used to clone TRBP C3 cDNA. TRBP C4 sense and TRBP C2 antisense were used to clone TRBP C4 cDNA. pGADGH-Dicer dsRBD (aa 1850–1922) was generated from pBluescript-Dicer-His6 [11] by PCR using 5' TCCCCCCGGGGAATGTACCCCGTTCCCCT-3' as sense primer and 5'CACGCGTCGACCTAGGGAACCTGAGGTTGATT-3' as antisense primer. The PCR fragment cut by *Xma*I and *Sal*I was subcloned in frame into pGADGH.

EGFP-C1-TRBP2 [30] and Myc-TRBP1 [25] were previously described. To construct Myc-TRBP2, the 5' end of TRBP2 was amplified by PCR using 5'-GCGGAATTCAGATGAGTGAAGAGGAG-3' as sense primer and 5'-CTAAGATCTTGTGCTTGGCTGCCTTC-3' as antisense primer. The amplified fragment cut by *Eco*RI and *Bgl*II was inserted in frame into pCMV-Myc (BD biosciences). The *Bst*EII-*Kpn*I fragment from pBS-TRBP2 [20] was inserted into this pCMV-Myc-5'end TRBP2 intermediate. To conscruct Myc-TRBP1ΔC4 and Myc-TRBP2ΔC4, a fragment of TRBP was amplified using the sense primer: 5'-GGATGCCCGGGATGGCAATGAGGTGGAGCCT-3' and antisense primer 5'-GCATGGTACCTCAGACACGGCAGCAGGCAGGG-3'. The amplified fragment was cut by *XmaI *and *KpnI *and inserted in frame into pCMV-Myc-TRBP1 or pCMV-Myc-TRBP2 cut with the same enzymes. The resulting proteins have 69 aa deleted in the terminal end of either TRBP1 or TRBP2. All inserts were verified by sequencing.

shRNA and miRNA expression cassettes against EGFP were constructed using a two step PCR method described previously [39]. In the first PCR step a partial expression cassette was amplified from pAVU6+27 [40] using the forward primer 5'-GGGCGCGGATCCAAGGTCGGGCAGGAAGAGGGCCT-3' and an appropriate reverse primer as follows:

Non-silencing shRNA:

5'-GTGGCTTCACAGACGTGACACGTTCGGAGAATCGCAGTATATGTGCTGCCGAAGCG-3' EGFP shRNA:

5'-GTGGCTTCACAGCTCCTTGAAGTCGATGCCCTCGCAGTCTCTGTGCTGCCGAAGCG-3' With the following thermal profile: 95°C for 5 min, 30 cycles (95°C for 1 min, 55°C for 1 min, 72°C for 1.5 min), 72°C for 7 min, using *Taq *polymerase (Invitrogen). The second PCR was performed with the matching primer as follows: Non-silencing miRNA: 5'-CTAGTCTAGAAAAAAAGCAGTTCTCCGAACGTGTCACGTCCCATCTGTGGCTTCACAGACGTG-3' EGFP miRNA:

5'-CTAGTCTAGAAAAAAAGCAGGGGCATCGACTTCAAGGAGCCCATCTGTGGCTTCACAGCTCCT-3' shRNA and miRNA expression vectors were generated by ligation of the final PCR product into pCR^®^II-TOPO^® ^(Invitrogen), and the resulting pCRII-U6+27 vectors confirmed by sequencing.

### Yeast two-hybrid assay

Yeast expression plasmids were introduced into the yeast reporter strain SFY526. The double transformants were selected and screened for β-galactosidase activity as described [25].

### Cells and transfections

Human HeLa and astrocytic cells U251MG, murine embryo fibroblasts (MEF) and primary tail embryonic fibroblasts *tarbp2*^+/- ^and *tarbp2*^-/-^, described in [26], were maintained in Dulbecco's modified Eagle's medium (DMEM; Invitrogen) supplemented with 10% fetal bovine serum (FBS) (Hyclone), 2 mM L-glutamine and 50 U/ml Penicillin and 50 ug/ml Streptomycin (Invitrogen).

### EGFP expression by fluorescence and FACS

Cells were transfected in 6-well plates with 4 μg sh- or mi-RNA constructs using TransIT Reagent (Mirus) at a 1:3 DNA:TransIT ratio prior to transfection with 1 μg pEGFP-C1 (Clontech). Cells were observed by fluorescence using an Axiovert 25 inverted microscope (Zeiss). EGFP expression was assed by calculating mean luminosity of individual fields, using Adobe Photoshop CS3 10.0.1 software. Results are expressed as a percent knock-down, as compared to non-silencing conditions. Cells were analyzed for EGFP expression by flow cytometry using a FACS Calibur cytometer (BD) and analyzed using Cellquest software (BD). Transfected cells were gated by green fluorescence greater than cells in a mock transfection. Relative fluorescence values were calculated as the product of the percentages of EGFP fluorescent cells gated and the mean fluorescence of cells gated positive.

### Immunoblotting

Cell lysates were prepared, separated and transferred for immunoblotting as previously described [25]. The membrane was blocked for 1 hr in 5% nonfat milk and 0.05% Tris-buffered saline 20 (TBST) [41]. The membranes were incubated overnight at 4°C with anti-TRBP 672 [26] at a 1/500 dilution, anti-EGFP and anti-Myc (Santa Cruz) antibodies at a 1/1000 dilution, or with anti-Dicer349 [11] at a 1/5000 dilution, in 5% milk/TBST. These were incubated for 1 hr at room temperature with a monoclonal anti-actin (Chemicon) at a 1/10,000 dilution. After 5 washes in TBST, membranes were incubated with peroxidase-conjugated secondary donkey anti-rabbit antibody (Amersham) for TRBP and Dicer, and sheep anti-mouse (Amersham) for EGFP, Myc and Actin at a 1/5000 dilution. The bands were visualized as previously described [38]. Bands were quantified by densitometry analysis, using Adobe Photoshop CS3 10.0.1 software.

### Co-immunoprecipitation (co-IP)

48 hrs post-transfection *tarbp2*^-/- ^cells were washed twice with PBS and lysed in cold lysis buffer [25] with protease inhibitors. For each IP, 40 μl of protein G sepharose fast flow compact beads (Sigma) were washed with TNEN (50 mM Tris-HCl [pH 7.4], 100 mM NaCl, 1 mM EDTA [pH 8], 0.5% NP40 (Sigma) and left rotating at 4°C for 4 hr with 2 μg anti-Myc (Santa-Cruz) or with 10 μl of monoclonal anti-Dicer 73 [11]. 1 mg of cell extract was added to the beads for overnight incubation at 4°C. The beads were washed 3 times with 1 ml of cold lysis buffer, 3 times with 1 ml of cold PBS, and resuspended in SDS loading dye. Bound proteins were eluted by boiling the beads for 10 min and fractionated by 7.5% SDS-PAGE. The immunoprecipitates were analyzed by immunoblot by using anti-TRBP 672, anti-Dicer, anti-Myc and anti-actin antibodies.

### Immunofluorescence

HeLa cells were cultured on coverslips in 12-well dishes. Untransfected cells were incubated with mouse anti-Dicer mAb42 [11] and rabbit anti-TRBP673 [18] antibodies. The cells were transfected at 50% confluency with Myc-TRBP1, Myc-TRBP2, Myc-TRBP1ΔC4, Myc-TRBP2ΔC4 or EGFP-TRBP2 using TransIT (Mirus) reagent. 24 hr post-transfection, the cells were washed three times with phosphate buffered saline (PBS) and fixed at room temperature for 20 min in PBS containing 4% paraformaldehyde and 4% sucrose. Cells were then washed three times in PBS, and permeabilized with 0.1% Triton X-100. The fixed and permeabilized cells were incubated in PBS containing 3% bovine serum albumin, (Sigma-Aldrich), 3% BSA-PBS. The cells were then incubated with mouse anti-Myc9E10 (ATCC), at a 1/500 dilution and rabbit anti-Dicer 349 [11] at a 1/250 dilution in 3% BSA-PBS at room temperature for 1 hr. The cells were washed 3 times with 3% BSA-PBS and incubated 1 hr with corresponding secondary antibodies: anti-rabbit Alexa Fluor 488 and anti-mouse Alexa Fluor 546 (Invitrogen, Molecular Probes™) at a 1/1000 dilution in 3% BSA-PBS. Cells were washed 5 times with PBS, rinsed with 95% ethanol, dried and mounted in Immu-Mount (Thermo-electron corporation). Cells were analyzed for fluorescence with a 65× objective on a confocal microscope (Zeiss LSM Pascal 5). Digital images were converted to TIFF format and compiled in Adobe PhotoShop CS3.

## List of Abbreviations

aa: amino acid; dsRNA: double-stranded RNA; dsRBP: dsRNA binding protein; dsRBD: dsRNA binding domain; EGFP: enhanced green fluorescent protein; FACS: fluorescence automated cell sorting; IF: immunofluorescence; IP: immunoprecipitation; Medipal: Merlin Dicer PACT liaison; MEF: murine embryonic fibroblast; miRNA: micro RNA; RISC: RNA-induced silencing complex; RNAi: RNA interference; shRNA: short hairpin RNA; siRNA: small interfering RNA; TEF: tail embryonic fibroblast; TRBP: TAR RNA binding protein

## Authors' contributions

SD performed the coIPs (Fig. 2), the fluorescence, the western blots and part of the FACS analysis (Figs. 4, 5, 6, 7). CMP set-up preliminary assays (Fig. 4, 6), performed the two-hybrid assays (Fig. 1) and part of the FACS analysis (Fig. 4, 6). RS performed the immunofluorescence (Fig. 1C and 3). AD designed and constructed several plasmids and provided technical advice. HC designed and constructed several plasmids. MEF designed and constructed several plasmids. DP supervised HC and provided input in the writing of the manuscript. SL performed some experiments with CMP, supervised him and provided input in the writing of the manuscript. AG designed, supervised all the work and wrote most of the manuscript. All authors read and approved the final manuscript.
